# An integrated finite element simulation of cardiomyocyte function based on triphasic theory

**DOI:** 10.3389/fphys.2015.00287

**Published:** 2015-10-20

**Authors:** Asuka Hatano, Jun-Ichi Okada, Takumi Washio, Toshiaki Hisada, Seiryo Sugiura

**Affiliations:** ^1^Department of Mechanical Engineering, School of Engineering, The University of TokyoTokyo, Japan; ^2^Department of Human and Engineered Environmental Studies, Graduate School of Frontier Sciences, The University of TokyoChiba, Japan

**Keywords:** triphasic theory, finite element method, cardiomyocyte, mitochondria, t-tubule

## Abstract

In numerical simulations of cardiac excitation-contraction coupling, the intracellular potential distribution and mobility of cytosol and ions have been mostly ignored. Although the intracellular potential gradient is small, during depolarization it can be a significant driving force for ion movement, and is comparable to diffusion in terms of net flux. Furthermore, fluid in the t-tubules is thought to advect ions to facilitate their exchange with the extracellular space. We extend our previous finite element model that was based on triphasic theory to examine the significance of these factors in cardiac physiology. Triphasic theory allows us to study the behavior of solids (proteins), fluids (cytosol) and ions governed by mechanics and electrochemistry in detailed subcellular structures, including myofibrils, mitochondria, the sarcoplasmic reticulum, membranes, and t-tubules. Our simulation results predicted an electrical potential gradient inside the t-tubules at the onset of depolarization, which corresponded to the Na^+^ channel distribution therein. Ejection and suction of fluid between the t-tubules and the extracellular compartment during isometric contraction were observed. We also examined the influence of t-tubule morphology and mitochondrial location on the electrophysiology and mechanics of the cardiomyocyte. Our results confirm that the t-tubule structure is important for synchrony of Ca^2+^ release, and suggest that mitochondria in the sub-sarcolemmal region might serve to cancel Ca^2+^ inflow through surface sarcolemma, thereby maintaining the intracellular Ca^2+^ environment in equilibrium.

## Introduction

Mathematical modeling has been used to further our understanding of the excitation–contraction coupling mechanisms of cardiomyocytes (Luo and Rudy, 1994; Jafri et al., 1998; Cortassa et al., 2006). Recently, three-dimensional (3D) cardiomyocyte models have been used to examine the effect of their intracellular structure on the reaction–diffusion process (Izu et al., 2001; Okada et al., 2005; Hatano et al., 2011, 2013b). In most of these studies, however, the intracellular electrical field and the fluid motion of cytosol were ignored on the grounds that their effects are negligible. Notwithstanding, as our focus approaches the microscale, with detailed and finely meshed 3D models (Hake et al., 2012), evidence has accumulated to suggest that those effects might not be negligible. Pasek and colleagues estimated the voltage drop along the t-tubule using cable theory (Pasek et al., 2008a). Their results showed that the membrane potential is likely to be uniform, except during the largest and fastest changes induced by physiological depolarization or by experimental voltage clamp. Heterogeneous distribution of ion channels between surface and t-tubular membrane (Pásek et al., 2008b) may also cause gradient of the membrane potential. Consideration of the electrical potential distribution is also important in diseased states such as Ca^2+^ wave-mediated membrane potential fluctuation (Fujiwara et al., 2008) and delayed after depolarizations (Wasserstrom et al., 2010), which are considered a cause of arrhythmias. McNary et al. (2011, 2012) modeled the stretching of a myocyte and predicted that the t-tubule would shorten and change cross-sectional shape, reducing volume. This led to the conclusion that t-tubule deformation can cause fluid exchange between the t-system and the extracellular space. The resultant convectional changes in ion concentration, even if small, can significantly affect the electrophysiology because the area of the cell membrane in the t-tubule is almost half that of the whole cell and is densely populated with various ion channels and transporters.

Triphasic theory integrates electrical field effects and fluid dynamics into the reaction–diffusion phenomenon and into the mechanical deformations involved in the simulation of cardiomyocyte excitation–contraction coupling. Triphasic theory is based on mixture theory, treating a solid phase, a fluid phase, and an ionic phase. It was initially developed to describe the deformation and stress fields for cartilage under chemical and/or mechanical loads (Lai et al., 1990). The conservation and conditional equations for each phase are solved simultaneously, which predicts the solid deformation, the fluid motion under pressure and osmotic forces, and the motion of ions by diffusion, convection, and electrical forces. Hirabayashi and coworkers used triphasic theory to model 2D cardiomyocyte excitation–contraction coupling (Hirabayashi et al., 2010; Okada et al., 2011) developed a 3D version by implementing parallelization algorithms.

We previously developed a 3D reaction–diffusion and contraction finite element model (FEM) of a cardiomyocyte that integrated electrophysiology, metabolism, and deformation (Hatano et al., 2011). This model was modified to incorporate triphasic theory to analyze the intracellular fluid dynamics, convective ion movement, and electrical gradient (Hatano et al., 2012a). We also increased computational efficiency by separating the updating of mechanical, electrical, and chemical variables into time steps appropriate for each phenomena (Hatano et al., 2013a). In this study, we present a triphasic-theory-based simulation using a 3D model of a quarter section of cardiomyocyte. First, we fitted parameters of the model against experimental results, and then examined the effects of t-tubule morphology and mitochondrial location on cardiac physiology.

## Materials and methods

The model is the triphasic extension of the model presented in Hatano et al. (2011), which is a reaction–diffusion finite element model of a guinea-pig cardiac ventricular cell at 37°C. The formulation and parameters for the reaction of subcellular components are the same as in the previous study, adapted from the ODE integrated guinea-pig model by Cortassa et al. (2006). See Hatano et al. (2011) and its supplemental material for further details.

The previous reaction–diffusion simulations reproduced the experimentally measured time changes of averaged ions, energy metabolites, and local Ca^2+^ concentrations (Hatano et al., 2011), Ca^2+^ propagation velocity in de-tubulated cardiomyocyte (Hatano et al., 2012b), and mitochondrial Ca^2+^ oscillation (Hatano et al., 2013b). The simulation results of the triphasic model were compared with that of the reaction–diffusion model, which were validated previously (Hatano et al., 2011).

### Structure of the 3D cardiomyocyte model

**Figure 1:**
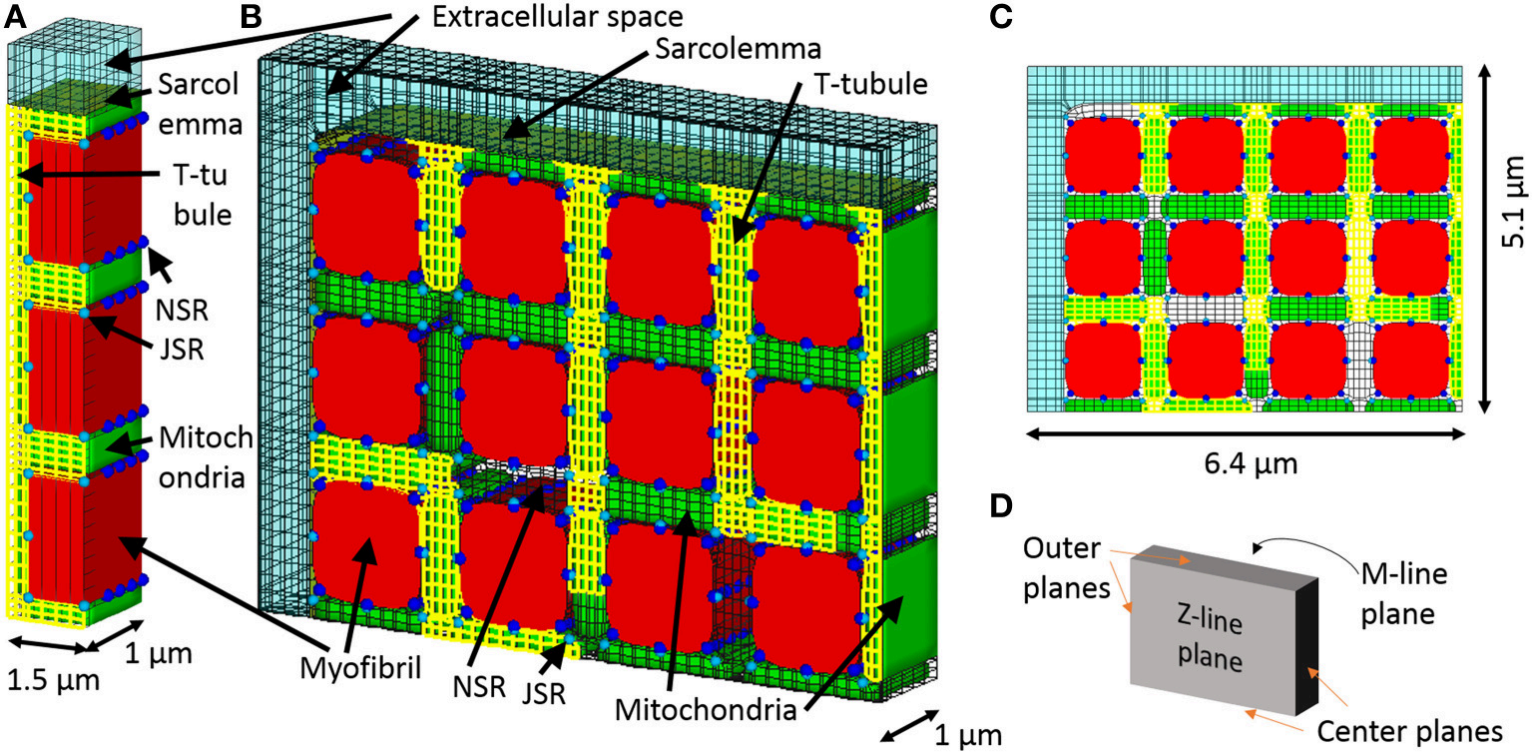
**3D model of a cardiomyocyte**. **(A)** The three myofibril mesh, which includes myofibril (red), mitochondria (green), network, and junctional sarcoplasmic reticulum (NSR and JSR, light blue, and blue respectively), sarcolemma (yellow), t-tubules (yellow), and the surrounding extracellular space (transparent light blue). **(B)** The quarter section mesh spans a half sarcomere in length and radially includes a quarter of the cross section. **(C)** Front view of the mesh. **(D)** Orthogonal reference planes seen from the same angle as **(A)**.

The three myofibril mesh, comprising a segment containing three myofibrils of half sarcomere length and the adjacent cell membrane and organelles, was used for validating and determining the diffusion coefficient inside the t-tubule, and the quarter cross-section half-sarcomere mesh was used for evaluating the roles of t-tubule morphology and mitochondrial location. The three myofibril mesh is the same as the mesh used in the reaction–diffusion simulations (Hatano et al., 2011), except for the addition of an extracellular space (Figure 1A). The intracellular space was filled according to the ratio myofibril: mitochondria: cytosol: junctional sarcoplasmic reticulum (JSR): network sarcoplasmic reticulum (NSR) = 54:35:8:0.03:3 (Cortassa et al., 2006). Longitudinal periodicity and symmetricity were assumed. The t-tubule is located on the z-line, avoiding the myofibril, NSR surrounds the myofibril, and JSR faces the t-tubule every ~0.4 μm (Chen-Izu et al., 2006). The model comprises 11067 nodes and 3837 elements. Ion channels, pumps, and exchangers are distributed on the surface sarcolemma and t-tubular membranes with specific densities, as previously reported for guinea pig ventricular myocyte (Pasek et al., 2008a). For more details, see (Hatano et al., 2011).

The quarter-section mesh is of half sarcomere length and spans one quarter of the cardiomyocyte cross-section, containing 12 myofibrils. We assume axial symmetry and longitudinal periodicity. The quarter section cardiomyocyte model is shown in Figures 1B–D. The t-tubules are located on z-lines, reproducing the structure visualized by fluorescent studies (Sachse et al., 2012). To determine the contribution of sub-sarcolemmal mitochondria and the effect of distance between myofibril and mitochondria on myocyte function, the mitochondria are located at random. Volume ratio, sarcoplasmic reticulum location, and ion channel distributions are the same as in the triphasic three myofibril model. The model comprised 105883 nodes and 29543 elements.

### A cardiomyocyte model integrating electrophysiology and mechanics based on triphasic theory

#### Governing equations

In triphasic theory, soft tissue, namely cardiomyocyte in this study, is assumed to be a mixture of three phases: (1) cytoskeletal structure as the solid phase, (2) cytosol as the fluid phase, and (3) mobile ions as the ionic phase. The three constituents are assumed to occupy simultaneously the same region. Mechanical and electrical balance equations and conservation equations are defined for each phase. We adopt the formulation of Okada et al. (2011); at each node, six variables (18° of freedom) are defined (Table 1), and the governing equations comprise the balance equation and the conditional equation for each phase. Herein, the superscripts s, w, and α respectively denote the solid, fluid, and ionic phases. The governing equations are as follows.

**Table 1:** **Variables and their numbers of degrees of freedom (DOF)**.

**Variable**	**DOFs**	**Description**
u	3	Displacement of solid phase
W	3	Fluid velocity relative to solid phase
P_S_	1	Pressure of solid phase
P_w_	1	Pressure of fluid phase
C^α^	9	Concentration of solute α (α = Na^+^, K^+^, Ca^2+^, Cl^−^, ATP, ADP, Cr, CrP, Pi)
Ψ	1	Electrical potential

The balance equation for the mixture is,

(1) $$\nabla_{\chi }\cdot \Pi =0,$$

which requires a compressibility condition for the solid phase,

(2) $$\text{J}-\text{1}+\frac{{\text{P}}_{S}}{\kappa_{S}}=\text{0}.$$

The balance equation for the fluid phase is

(3) $$\nabla_{\chi }{\text{P}}_{w}+\text{RT}(\text{1}-\varphi )\nabla_{\chi }{\text{C}}^{\text{total}}+{\text{K}}^{-1}W-{\text{C}}^{\text{F}}\nabla_{\chi }\Psi =0$$

Above, after the pressure gradient (first term), we have the osmotic pressure (second term), the frictional force in the solid phase (third term), and the effect of the electrical potential gradient (fourth term). We now define an incompressibility condition for the mixture, such that a change in mixture volume equals outflow of fluid,

(4) $$\text{J}-\text{1}+\nabla_{\chi }\cdot Q^{w}=0,$$

(5) $$Q^{w}\equiv \int_{0}^{\text{t}}W\text{dt}.$$

The balance equation for the ionic phase resolves electrical and frictional forces on ions with ion conservation,

(6) $$\begin{array}{l} {Ċ}^{\alpha }\Phi^{\text{w}}-(\nabla_{\chi }{\text{C}}^{\alpha })\cdot W+\nabla_{\chi }\cdot {\text{D}}^{\alpha }\left(\nabla_{\chi }{\text{C}}^{\alpha }+\frac{{\text{z}}^{\alpha }{\text{F}}_{\text{c}}}{\text{RT}}{\text{C}}^{\alpha }\nabla_{\chi }\Psi \right)\\ \;-f_{\text{subcell}}^{\alpha }=0. \end{array}$$

The change in ion concentration (first term) is equilibrated with the ion flux due to convection (second term), diffusion (third term), electrical forces (fourth term), and subcellular reactions (fifth term).

Finally, the electroneutrality condition is

(7) $$\sum_{\alpha }{\text{z}}^{\alpha }{Ċ}^{\alpha }+{Ċ}^{\text{F}}=\text{0};$$

**Π** is first Piola-Kirchhoff stress of mixtures, J is the volume change (determinant of the deformation gradient tensor), χ is the reference configuration, κ_S_ is the volume modulus of the solid phase, C^total^ is the concentration of all the ions, C^F^ is the concentration of the charges captured by subcellular components and buffers, **z** is the valence of the ions, Φ^w^ is the fluid volume fraction, D^**α**^ is the diffusion coefficient of the solute α, K is the coefficient of permeability, **φ** is the osmotic coefficient, R is the gas constant, and T is the absolute temperature.

We use the subcellular reaction functions from our previous 3D reaction–diffusion–mechanical model of the cardiomyocyte (Hatano et al., 2011), which corresponds to $f_{\text{subcell}}^{\alpha }$ in Equation (6). The uptake and release of ions by subcellular components is affected by the local ion concentrations, which affect cell-wide concentrations through convection, diffusion, and electrical transport. The new parameters are described in Table 2, while all other parameters were the same as in the previous model (Hatano et al., 2011).

**Table 2:** **Parameter values for the triphasic model**.

**Parameter**	**Value**	**Unit**	**Description**
D^K^	980	μm^2^/ms	Diffusion coefficient of K^+^ in cytosol^[a^^,^ ^b]^^*^
D^Na^	665	μm^2^/ms	Diffusion coefficient of Na^+^ in cytosol^[a^^,^ ^b]^^*^
D^Cl^	1015	μm^2^/ms	Diffusion coefficient of Cl^−^ in cytosol^[a^^,^ ^b]^^*^
K	700	μm^4^/mN/ms	The coefficient of permeability (fitting parameter)
κ_S_	25	kPa	Bulk modulus of solid phase (fitting parameter)
G	1.0	μm^3^/MPa/ms	Conductance of triphasic truss element (fitting parameter)
C	100 × vol_t−elem_	μm^3^/MPa	Capacitance of triphasic truss element (fitting parameter)
ϕ${}_{\text{mitochondria}}^{\text{w}}$	0.617	–	The fluid volume fraction in mitochondria^[c]^
ϕ${}_{\text{myofibril}}^{\text{w}}$	0.787	–	The fluid volume fraction in myofibril^[c]^
ϕ${}_{\text{SR}}^{\text{w}}$	0.752	–	The fluid volume fraction in SR^[c]^
ϕ${}_{\text{cytosol}}^{\text{w}}$	0.833	–	The fluid volume fraction in cytosol^[c]^
ϕ${}_{\text{t}-\text{tubule}}^{\text{w}}$	0.900	–	The fluid volume fraction in t-tubule (fitting parameter)

[a] *Mori et al., 2008*.

[b] *Kushmerick and Podolsky, 1969*.

[c] *Aliev et al., 2002*.

* *Diffusion coefficients in cytosol are reduced to half of those in water (Kushmerick and Podolsky, 1969)*.

#### Model of sarcolemma

As the sarcolemma is thin compared with the resolution of our model, we ignored its thickness and applied capacitance approximation as for the prior model (Okada et al., 2013). At the node on the sarcolemma, there are two degrees of freedom assigned to the intracellular and extracellular regions (suffixes i and e, respectively). The electrical potential on the sarcolemmal node is calculated through the electroneutrality condition, taking into account ion currents and capacitive current. At the intracellular and extracellular sarcolemmal node, the respective electroneutrality conditions are described as follows:

(8) $$\sum_{\alpha }{\text{z}}^{\alpha }{Ċ}_{\text{i}}^{\alpha }\Phi^{w}+{Ċ}_{\text{i}}^{\text{F}}\Phi^{w}+\frac{{\text{I}}_{\text{total}}}{\text{F}}+\frac{{\text{C}}_{\text{m}}}{\text{F}}({\dot{\Psi }}_{i}-{\dot{\Psi }}_{e})=\text{0},$$

(9) $$\sum_{\alpha }{\text{z}}^{\alpha }{Ċ}_{\text{e}}^{\alpha }\Phi^{w}+{Ċ}_{\text{e}}^{\text{F}}\Phi^{w}-\frac{{\text{I}}_{\text{total}}}{\text{F}}-\frac{{\text{C}}_{\text{m}}}{\text{F}}({\dot{\Psi }}_{i}-{\dot{\Psi }}_{e})=\text{0}.$$

Sarcolemmal potential is evaluated at each node as Ψ_*i*_ − Ψ_*e*_ in the present model. At the same time, in the previous reaction–diffusion simulation, sarcolemmal potential was lumped throughout the model, therefore a change in potential is given as the sum of ion currents of all sarcolemmal nodes,

(10) $$\frac{\text{d}\Delta \Psi }{\text{dt}}=-\frac{1}{{\text{C}}_{\text{m}}}\frac{\sum_{\text{cell}}{\text{I}}_{\text{total}}}{\sum_{\text{cell}}\text{area}},$$

where area refers to the area of the sarcolemma and Σ_cell_ is the sum over the cell.

#### Triphasic truss element

The t-tubule structure is modeled using simplified triphasic truss elements to reduce computational cost. The mechanical formulation uses the general truss element, and the balance and conditional equations for the ion phase are the same as those for the solid element. Fluid is driven by the pressure difference across the edges of the truss element. Therefore, fluid velocity is no longer an independent variable. The fluid velocity in the truss element connecting nodes *i* and *j* is given by *W*_*j*→*i*_ = G(*p*_*j*_*-p*_*i*_), where *p*_*i*_ is pressure at node *i* and G is the conductance. Fluid conservation at node *i* is given by

(11) $$\sum_{j}W_{j\to i}=\sum_{j}G(p_{j}-p_{i})=0.$$

At the open end of the t-tubule, fluid exchange between the truss element and solid element (extracellular space) follows

(12) $$\int N_{i}(J-1+\nabla_{\chi }\cdot Q^{w})dV=\int_{0}^{t}\sum_{j}G(p_{j}-p_{i})dt.$$

On other nodes, the t-tubule facing the intracellular solid dilates according to the pressure difference. Volume conservation at node *i* is written as follows,

(13) $$\int N_{i}(J-1+\nabla_{\chi }\cdot Q^{w})dV=\int_{0}^{t}C\frac{\partial (p_{j}-p_{i})}{\partial t}dt,$$

where C is the volume compliance of the t-tubule.

### Parameter fitting

We used the three-myofibril model to evaluate the significance of triphasic theory by comparing with our reaction–diffusion simulation findings. Using the same model parameters, the equivalent mesh, boundary conditions for isometric contraction and the initial conditions for the steady state in the 1 Hz reaction–diffusion simulation, a 1 s excitation–contraction cycle was simulated and compared. In the reaction–diffusion model, the extracellular space was not meshed as it was assigned constant bulk extracellular ion concentrations. In the triphasic model, ion concentrations on the nodes at the boundary of the extracellular space were fixed at the same constant values. We used their steady-state values from the reaction–diffusion model operating at 1 Hz as the initial conditions for the triphasic model to save the computational time.

To determine the diffusion coefficient in the t-tubule, the simulation predictions of ion diffusion in t-tubules and the resultant changes in membrane currents were compared with the experimental data of Swift et al. (2006) and Shepherd and McDonough (1998). When an isolated cell is exposed to rapid changes in extracellular ion concentration, the variations in intra-t-tubule ion concentration are delayed because of diffusional restriction. The diffusion coefficient inside the t-tubule can be estimated by comparing the variation in whole-cell membrane currents with extracellular ion concentration in experiments to that in simulation by applying a particular diffusion coefficient to the interior of the t-tubule.

Swift et al. (2006) measured changes in current and membrane potential in control and osmotically de-tubulated cardiomyocytes during rapid changes in extracellular potassium concentration [K^+^]_o_ (from 5.4 to 8.1 mM) under voltage-clamped (−80 mV) control. Shepherd and McDonough (1998) recorded changes in peak calcium current (I_Ca_) caused by rapid changes in the extracellular calcium concentration ([Ca^2+^]_o_) (from 0.45 to 1.8 mM) in voltage-clamped (−45 mV) guinea pig ventricular cardiomyocyte at various intervals prior to the activation of I_Ca_.

First, we simulated the experiment by Swift et al. (2006) by changing extracellular K^+^ concentration ([K^+^]_o_) from 5.4 to 8.1 mM under voltage clamped (−80 mV) conditions while applying various diffusion coefficients to the interior of the t-tubule. We then selected the diffusion coefficient value that gave the best fit to the experimentally observed time course of membrane current. Using this value, we simulated the experiment by Shepherd and McDonough (1998), in which [Ca^2+^]_o_ was switched from 0.45 to 1.8 mM with voltage clamped to −45 mV. The time course of the whole cell I_Ca_ was recorded in response to a step change in voltage to 0 mV applied at various intervals (25, 65, 85, 185, 485 ms). To simulate the voltage clamp protocol, we fixed the electrical potential of the nodes at the boundary of the extracellular space to zero. The electrical potential of the nodes on the M-line in the cell core were fixed at the intended voltage (−80, −45, or 0 mV). To simulate the experiments of Swift et al. (2006), we made a model without t-tubules based on the three myofibril mesh.

### Boundary conditions and simulation protocol

For electrical and ionic calculation, we apply symmetry boundary conditions at the center, Z-line, and M-line planes, as indicated in Figure 1D. To model extracellular bulk solution, solute concentrations are fixed at initial bulk concentrations and electrical potential is fixed at zero on the outermost plane. For mechanical calculations, a symmetry boundary is also applied on the center planes. For isometric contraction, the nodes along the Z-line and M-line are fixed on the plane. For free contraction, the nodes along the Z-line are fixed on the plane and the nodes along the M-line have a prescribed planar motion, which is realized by introducing a Lagrange multiplier on their perpendicular degree of freedom.

Because the dynamics of ionic and electrical phenomena limit the time step of each phase, we separate mechanical, electrical, and chemical variables and update them with different time steps (1 ms, 10^−2^ ms, adaptive between 10^−2^ and 10^−5^ ms, respectively), which assures their individual convergence (Hatano et al., 2013a). Depolarization is triggered by applying a stimulation current of −100 A/F for 0.5 ms to all the sarcolemmal nodes.

All software was written in-house using the Fortran programming language. Computation was performed using an Intel (Santa Clara, CA, USA) Xeon CPU (2.6 GHz). The calculation took 150 h for 1 s simulation of the quarter model.

## Results

### Comparison with previous experimental model

**Figure 2:**
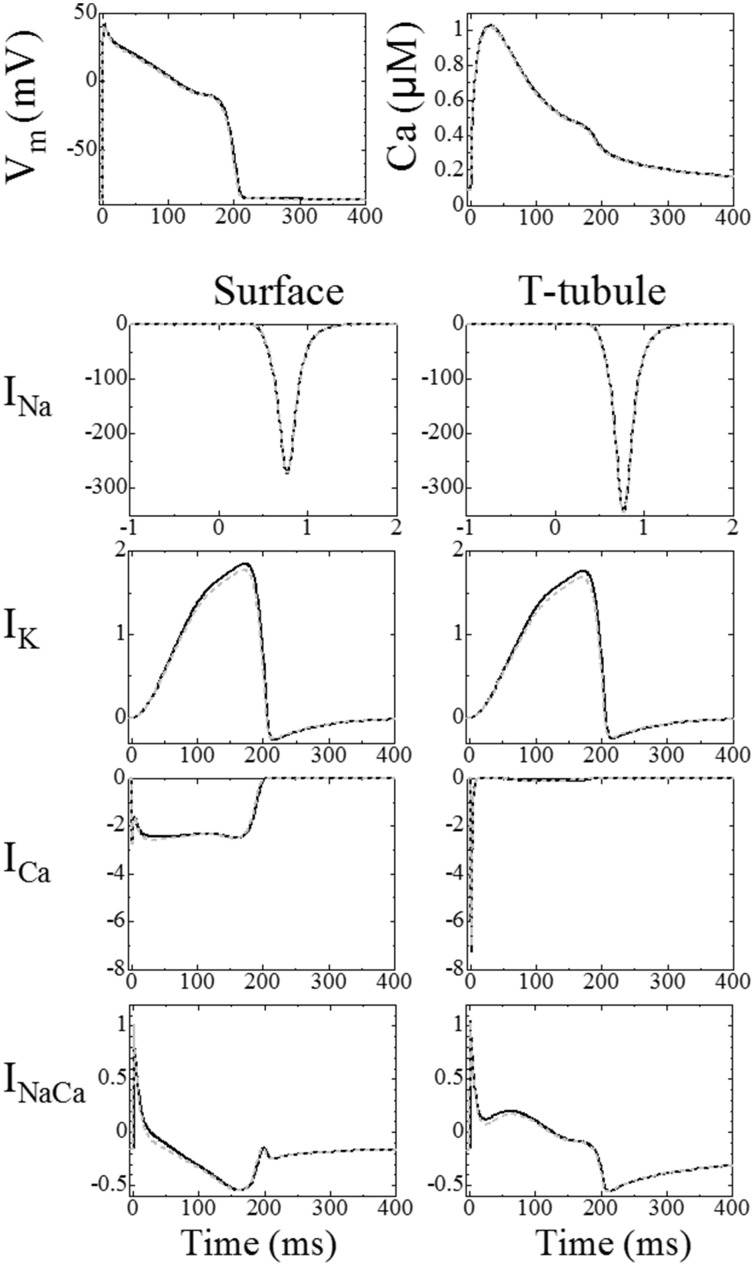
**Comparison between the triphasic model (black solid line) and reaction–diffusion model calculated without an electrical potential gradient (gray broken line)**. **Top left:** mean membrane potential; **top right:** mean Ca^2+^ concentrations. The **bottom** eight panels show ion currents (A/F), the left column shows the current through the surface membrane and the right column shows the current through the t-tubular membrane [reproduced from Hatano et al. (2013a) by copyright permission of Japanese Society for Medical and Biological Engineering].

A comparison of the triphasic model with the prior reaction–diffusion model was previously reported in Japanese (Hatano et al., 2012a). The results of this study are summarized below. On the basis that our previous reaction–diffusion simulation has already been validated and that triphasic extension is thought to have little effect on the averaged responses of the model, the triphasic simulation results were compared with the reaction–diffusion simulation results using the same three myofibril mesh. Figure 2, reprinted from Hatano et al. (2012a), shows the mean membrane potential, mean Ca^2+^ concentrations and membrane currents of the triphasic simulation (solid line) and the reaction–diffusion–contraction simulation (gray broken line). We can see that general electrophysiological phenomena are reproduced by the triphasic model. The differences in the concentrations of the ions and metabolites between the two simulations were less than 1%. The contributions of the electrical and convectional terms on the change of intracellular ion concentrations were 2 and 0.02%, respectively, in the maximum compared with the diffusional term.

**Figure 3:**
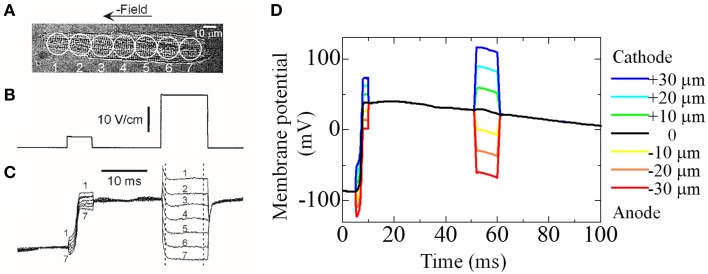
**Experiment and simulation of electrical field induced depolarization**. **(A–C)** Experimental result reproduced from Sharma et al. (2002), by copyright permission of IEEE. **(A)** Cardiac cell that was stimulated with field pulses of 5 and 23 V/cm shown in **(B)**. The responses recorded from seven sites along the cell length are shown in **(C,D)** Simulated responses of membrane potential at seven sites to the field pulses of 12 mV (5–10 ms) and 30 mV (50–60 ms) [reproduced from Hatano et al. (2012a) by copyright permission of the Japan Society of Mechanical Engineering].

We previously reported the significance of triphasic theory in simulating the electrophysiology of cardiomyocytes (Hatano et al., 2013a) when compared with experimental findings of Sharma et al. (2002). We prepared a whole cell but coarser mesh for this simulation. Sharma et al. (2002) applied field stimulation of a variable magnitude (Figure 3B) and recorded the membrane potential at seven sites aligned longitudinally along the cell (Figure 3A). They found that field stimulation applied to the resting cell evoked gradual depolarization, although the potential was more positive at the cathode side, suggesting that an inward current on this side caused a potential distribution along the cell. Our simulation, which mimicked this protocol, reproduced these potential distributions with the inward current flowing from cathode to the anode side (Figures 3C,D). These findings suggest that a potential distribution exists on the cellular scale, a phenomenon that can be reproduced by the model based on triphasic theory (Hatano et al., 2013a).

### Determination of diffusion coefficient inside of t-tubule

**Figure 4:**
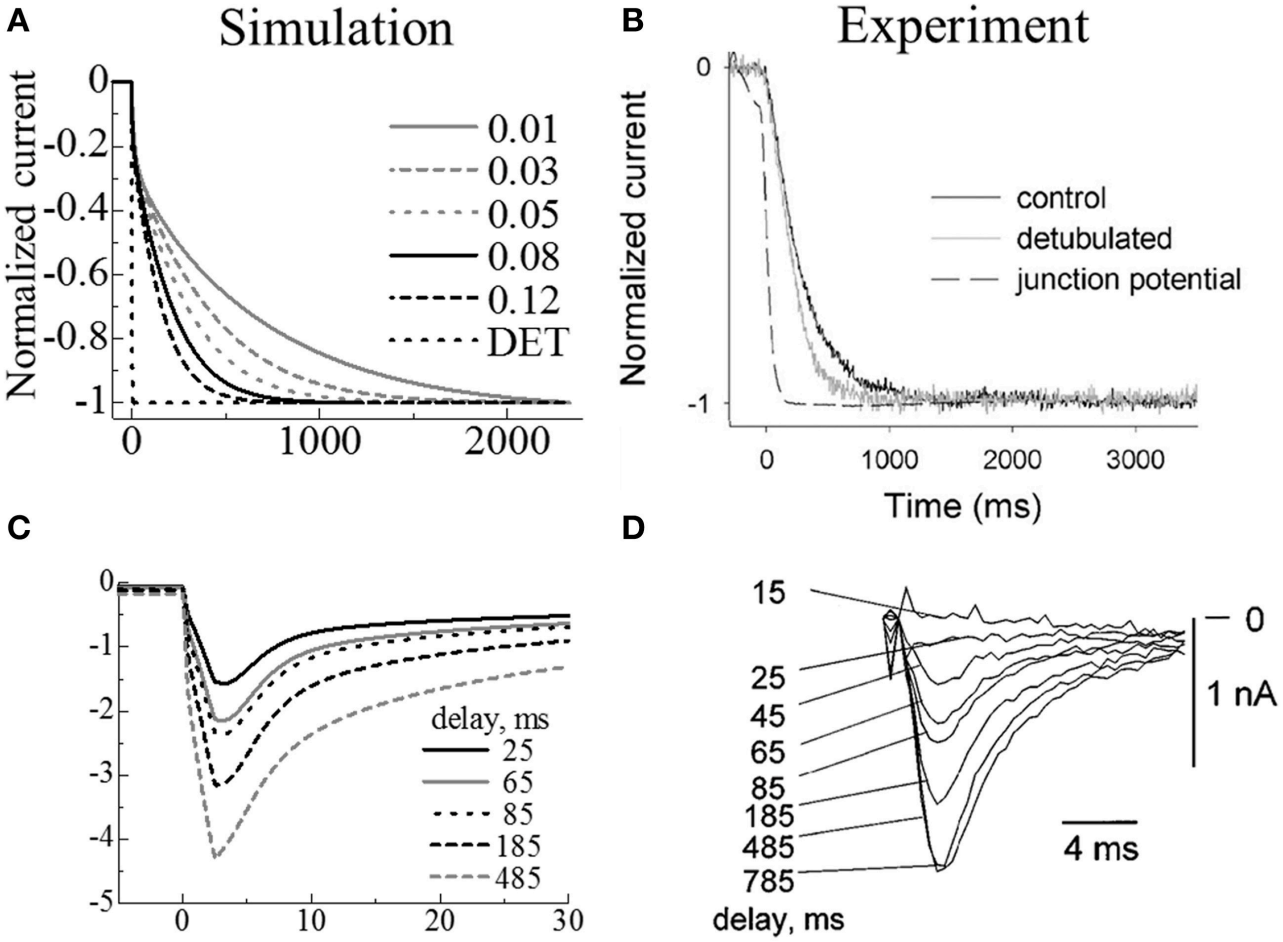
**Simulated membrane currents following changes in extracellular ion concentrations (A,C) compared with experimental results (B,D)**. **Upper panels:** Membrane currents when extracellular K^+^ increased from 5.4 to 8.1 mM. **(A)** Simulated results using models with various intra-t-tubule diffusion constants and a model without t-tubules (DET); the legend values are rates of applied diffusion constant inside the t-tubules relative to those of cytosol. **(B)** Reprint of experimental measurements by Swift et al., using control (black) and de-tubulated (gray) myocytes. (Copyright permission by American Physiological Society). **Lower panels:** I_Ca_ current on activation after a certain delay in increasing the extracellular Ca^2+^ from 0.45 to 1.8 mM. **(C)** Simulated results. **(D)** Reprint of experimental measurements by Shepherd et al. (Shepherd and McDonough) (Copyright permission by American Physiological Society).

We simulated Swift et al.'s (2006) experiment using the simple three-myofibril model. The appropriate intra-t-tubule diffusion constant to reproduce the experimental results was determined by a sensitivity study. Figure 4A shows the temporal change of normalized membrane currents after changing extracellular K^+^ concentration ([K^+^]_o_) from 5.4 to 8.1 mM under voltage clamped conditions. The legend values are rates of applied diffusion constant inside the t-tubules relative to those of cytosol. The diffusional restriction delayed changes in the intra-t-tubule [K^+^], which also caused gradual changes in the membrane currents. In Figure 4B, we compare the numerically predicted times to reach 50, 90, and 95% of the new steady-state current with those from the experiments. The [K^+^] diffusion constant of 72 μm^2^/s, corresponding to 0.08 times the cytosolic value and 0.04 times the aqueous phase (Mori et al., 2008), had the best fit with experimental data. This is similar to the value estimated by Swift et al. (85 μm^2^/s) using a one-dimensional simulation.

In the experiment, the membrane current of the de-tubulated myocyte changed gradually. This is in marked contrast to our simulation predictions of almost instantaneous variation (Figures 4A,B). The reason for the gradual change observed in experiments remains unclear, but as discussed in the original paper (Swift et al., 2006), incomplete de-tubulation and existence of caveolae may have slowed the diffusion, or coverslips used in the experiment may have restricted the exchange of extracellular solution. In our simulations, we assumed complete de-tubulation and the instantaneous change of ionic concentrations in the extracellular solution. Therefore, our de-tubulated model experienced no delay in changing in membrane current (DET in Figure 4A).

We used the parameter values from our simulations of the Swift experiments in the subsequent simulations of the Shepherd experiments. Figures 4C,D compare the simulation predictions and experimental recordings of I_Ca_ current during step changes in voltage from −45 to 0 mV for several step intervals (25, 65, 85, 185, 485 ms) after changing extracellular [Ca^2+^]_o_ from 0.45 to 1.8 mM. As the timing was prolonged, the maximum inward current progressively increased, thus showing good agreement with the experimental results.

### Effect of t-tubule distribution on Ca^2+^ dynamics

**Figure 5:**
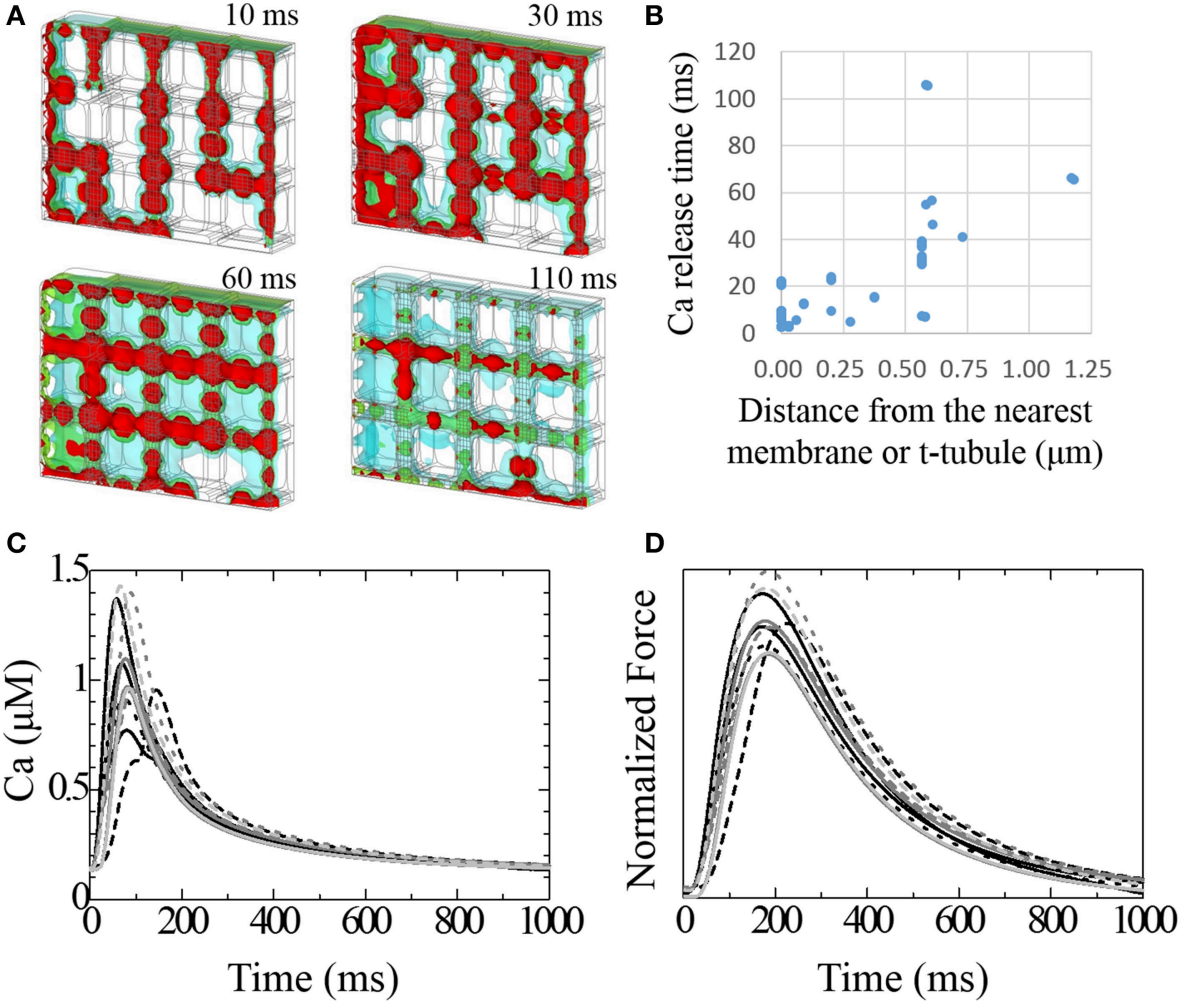
**Transient responses of Ca^2+^ and developed force**. **(A)** Time lapse image of intracellular [Ca^2+^] seen from the same mesh angle as in Figure 1A. [Ca^2+^] isosurfaces (4.0, 2.5, 1.0 mM; red, green blue, respectively) at 10, 30, 60, and 110 ms after stimulation. **(B)** The relationship between the delay of Ca^2+^ release after stimulation and the distance from the nearest membrane or t-tubule for each JSR. **(C)** Transient change in [Ca^2+^] at the center of each myofibril. **(D)** Normalized developed force at the center of each myofibril.

Figure 5A shows time lapse images of the distribution of intracellular calcium concentration ([Ca^2+^]_i_) after the onset of excitation (see also Supplemental Movie 1). [Ca^2+^]_i_ begins to increase near the t-tubule and sarcolemma because of the L-type Ca^2+^ channel (LCC) current, which triggers the Ca^2+^ release from nearby JSR; diffusion of Ca^2+^ provokes Ca^2+^ release from the remote JSRs. The relationship between delays in the timing of Ca^2+^ release in these JSRs and the distance from the nearest t-tubule or sarcolemma is shown in Figure 5B. The JSR facing the t-tubule or sarcolemma releases Ca^2+^ within 20 ms. This delay was strongly correlated with the distance in most cases. The JSRs exhibiting a larger delay are located in the bottom-right region (Figure 5A). This part of the model is characterized by a lack of mitochondria. The significance of this finding will be discussed later.

Varying the timing of the JSR Ca^2+^ release produced a spectrum of local Ca^2+^ transients. Figure 5C shows the local Ca^2+^ transients measured at the center of each myofibril. The earliest peak in the Ca^2+^ transient among the 12 myofibrils occurred 57 ms after depolarization, and the latest occurred after 144 ms. The Ca^2+^ transient profile in the myofibril next to the most recently excited JSR exhibited early and late peaks. Three myofibrils located in the sub-sarcolemmal region but without sub-sarcolemmal mitochondria (SSM) exhibited larger amplitudes than the others. The difference in peak timing of force development among the myofibrils was less conspicuous compared with that of Ca^2+^ transient (earliest 171 ms, latest 225 ms). Nonetheless, the third sub-membrane myofibril produced an approximately 15% larger force amplitude than the others (Figure 5D).

### Fluid movement in t-tubules

**Figure 6:**
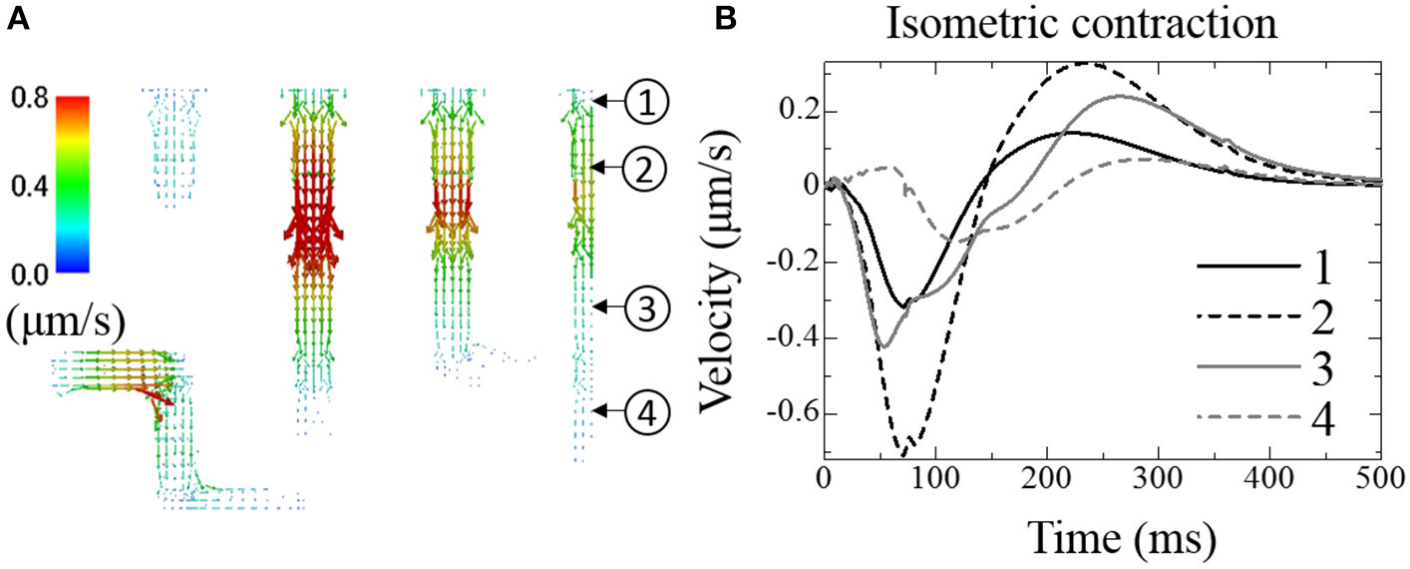
**Fluid exchange between extracellular space and t-tubules under isometric contraction**. **(A)** Distribution of fluid velocity 100 ms after depolarization. **(B)** Transient change in fluid velocity at the location indicated in **(A)**. Outward flow from the t-tubule (core to surface) is defined as positive.

Figure 6A shows the distribution of fluid velocity under isometric contraction. (See Supplemental Movie 2 for the temporal changes in distribution with the same color scale as Figure 6A) The temporal changes of fluid velocity at four points along the t-tubule located in the center plane are shown in Figure 6B. The contraction force developed under isometric conditions produced a negative pressure within the cell, expanding the t-tubules and allowing extracellular solution to flow into the t-tubules. Upon relaxation, the unperturbed t-tubule volume was recovered, thereby squeezing out the fluid. Because the t-tubule diminishes in diameter from the inlet, the velocity is smaller at location 1 (inlet). Delayed Ca^2+^ release and force development near location 4 resulted in a phase delay of fluid motion in this location.

### Distribution of electrical potential

**Figure 7:**
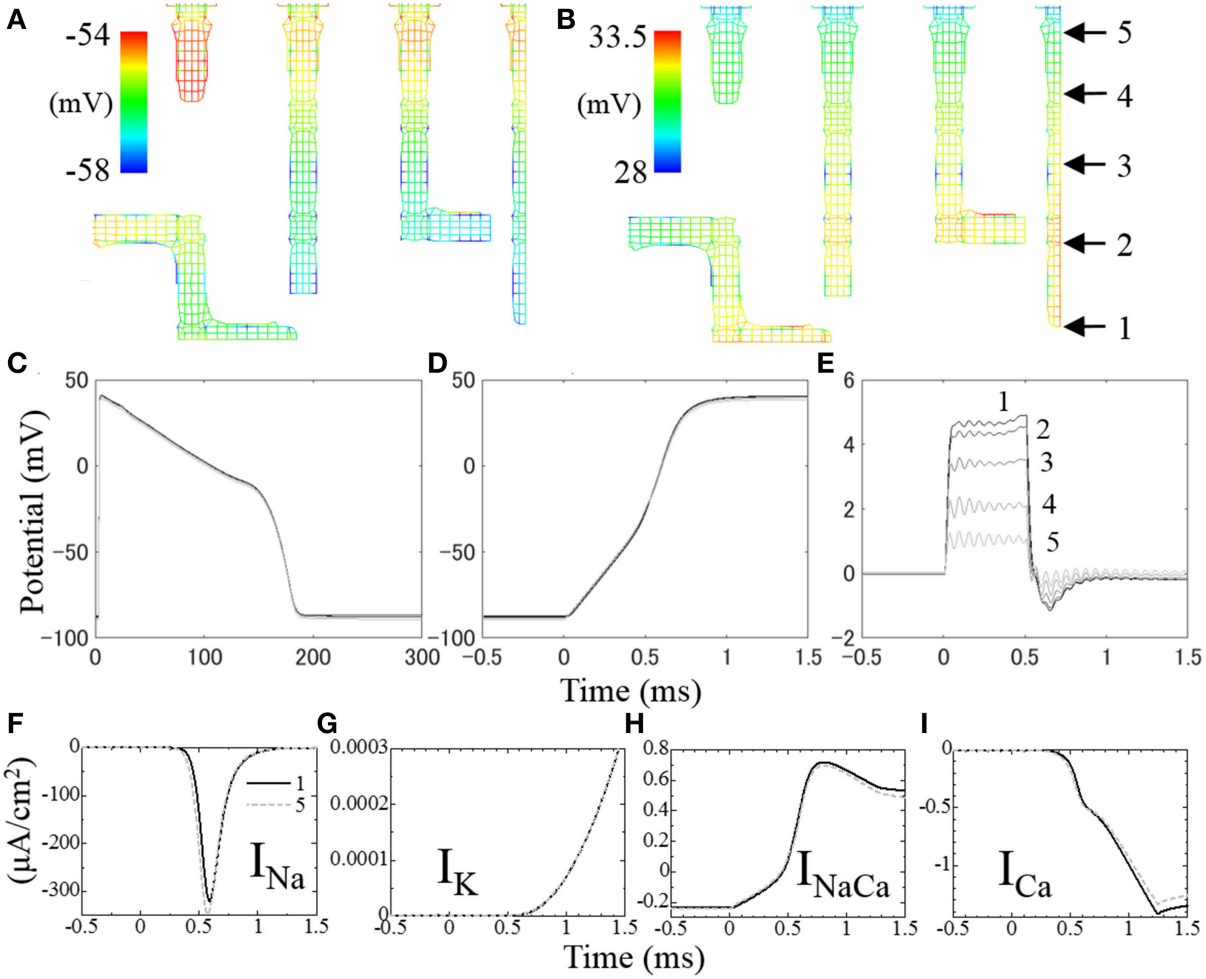
**Distribution of membrane potential in t-tubules at (A) 0.3 ms and (B) 0.6 ms after the onset of stimulation**. The temporal changes of membrane potential along the t-tubule on the center plane (indicated in **B**) are shown in **(C–E)**. **(C)** Membrane potential for 300 ms, **(D)** intracellular electrical potential for the first 2 ms at the onset of depolarization, **(E)** extracellular (inside of t-tubule) electrical potential for the first 2 ms. **(F–I)** Local ion currents for the first 2 ms at the locus 1 (solid line) and locus 5 (broken line) indicated in **(B)**.

Figures 7A,B show membrane potential distributions at 0.3 and 0.6 ms after the onset of stimulation, respectively. During the first 0.5 ms, a stimulation current is applied to the surface of the sarcolemma causing a rise in potential to propagate from surface to core (Figure 7A). After 0.5 ms, the activated Na^+^ current depolarizes the cell membrane (Figure 7B). As we distributed more Na^+^ channels in the t-tubules than in surface sarcolemma, following Pásek et al. (2008b), the rise in membrane potential was faster in the t-tubules (Figure 7B).

The spatio-temporal distribution of membrane potential along the t-tubules is shown in Figure 7C. Significant distribution was observed at the timing of depolarization, which is in agreement with cable theory (Pasek et al., 2008a). Figure 7D shows the spatio-temporal distribution of intracellular electrical potential along the length of the t-tubules, and Figure 7E shows the equivalent for the intra-t-tubules (extracellular spaces). The largest potential difference was observed at depths of the intra-t-tubule, which peaked during 0.5 ms of applied stimulation current, and was lowest at the peak of Na current. Membrane potential reflects the difference of electrical potential across the cell membrane, and its spatial distribution is mainly caused by the distribution of intra-t-tubular potential due to the much smaller distribution of intracellular potential. The higher intra-t-tubular potential gradient is created by the limited diffusion in this space. Such limited diffusivity causes high resistivity for ion movement, creating a larger potential difference compared with the intracellular space, where ions move more freely (Hatano et al., 2012a).

Ion currents at locus 1 at depths of the t-tubule (solid line) and at locus 5 near the surface (broken line) are shown in Figures 7F–I, with the loci indicated in Figure 7B. Because of the delay in membrane potential at deep regions, the voltage-gated Na channel opens late and the peak I_Na_ value was ~8% smaller compared with that near the surface. There was also a slight difference in other t-tubule ion currents (I_K_, I_NaCa_, and I_Ca_) between the deep and surface regions during the first 0.8 ms of action potential. However, the magnitudes of these currents were small at this time. The more marked differences in I_NaCa_ and I_Ca_ observed after 0.7 ms were not homogeneous along the t-tubule due to the difference in intracellular [Ca^2+^] caused by the distance from the Ca^2+^ release unit.

## Discussion

### Confirmation of the triphasic extension

In our previous study, we validated our reaction–diffusion–contraction finite element model by reproducing the variation in timing of the averaged membrane potential, the concentrations of the ions and metabolites, the local Ca^2+^ distribution (Hatano et al., 2011), the Ca^2+^ propagation in the de-tubulated cell (Hatano et al., 2012b), and the mitochondrial Ca^2+^ oscillation (Hatano et al., 2013b). As changes in general responses introduced by the triphasic extension were supposed to be small, we assumed that the validation was still valid therefore we compared our triphasic electrophysiological–mechanical model against the reaction–diffusion-contraction finite element model in our previous paper (Hatano et al., 2012a). The triphasic model predicted the averaged membrane potential, ionic concentrations, subcellular component states, and shortening deformation measured in the experiments. In the present study, we first performed simulations mimicking two experimental protocols to find that an intra-t-tubular diffusion coefficient of 0.08 times the cytosolic value can reproduce the empirical results. We used this diffusion coefficient rate for all ions (K^+^, Na^+^, Ca^2+^, Cl^−^) as several studies estimated similar values for different ions (Yao et al., 1997; Blatter and Niggli, 1998; Shepherd and McDonough, 1998; Swift et al., 2006). Furthermore, we presumed that the diffusion barrier was more likely to be a common mechanism involving the chemical properties of individual ion species. In our simulations, this single value reproduced the experimental findings of the delayed whole cell ion currents following a rapid change in both [K^+^] and [Ca^2+^] in the extracellular solution (Shepherd and McDonough, 1998; Swift et al., 2006).

The triphasic extension of the model successfully reproduced the field stimulation-induced depolarization reported by Sharma et al. (2002). Our finding of normal contraction suggested the existence of a potential distribution of ~5 mV at the onset of depolarization, resulted in an ~8% difference in the peak Na current. However, the time scale of this phenomenon was within milliseconds, and had minimal effect on cellular dynamics under normal conditions, which supports the use of the lumped membrane potential approximation adopted in the majority of previous models. However, the integration of the spatio-temporal distribution of electrical potential is crucial when we consider abnormal conditions, such as Ca^2+^ wave-induced depolarization (Fujiwara et al., 2008; Wasserstrom et al., 2010). Further applications of triphasic theory to subcellular phenomena involving the electrical-physiology are required. In addition, the role of voltage distribution becomes increasingly important when simulating larger scale dynamics, such as simulation of excitation propagation in cardiac tissue (Okada et al., 2013). The use of triphasic theory may allow seamless simulation of cardiac electrophysiology from the subcellular to organ scale.

### Effect of t-tubule and mitochondrial morphology on Ca^2+^ dynamics

In the present study, we improved our modeling capability by extending the segment to be half a sarcomere in length and spanning quarter of the cross section to contain 12 myofibrils. The larger model enabled us to include randomness in the t-tubule morphology and in the mitochondrial location. Our results demonstrated that a delay in the release of Ca^2+^ from the JSRs increases in proportion to the distance from the sarcolemmal or t-tubule membrane. This confirms previous experimental and modeling finding that the well-developed and dense t-tubule structure is important for synchrony of Ca^2+^ release (Louch et al., 2004, 2010; Cheng et al., 2010; Hatano et al., 2012b; Øyehaug et al., 2013).

We also found that among JSRs distant from the membrane, those located adjacent to mitochondria released Ca^2+^ the fastest. Cytosolic [Ca^2+^] surrounding the mitochondria is maintained at a higher level at rest and at a lower level during excitation compared with [Ca^2+^] at loci without mitochondria, as mitochondrial Ca^2+^ exchange occurs through uniporters and Na^+^–Ca^2+^ exchangers. This higher [Ca^2+^] under resting conditions facilitate the Ca^2+^ release from JSR adjacent to mitochondria.

Mitochondria play another role in Ca^2+^ dynamics in the sub-sarcolemmal region. The myofibrils next to sarcolemma but without SSM were exposed to higher peak Ca^2+^ than the other myofibrils. At the myofibrils next to the sarcolemma, Ca^2+^ flows in from the sarcolemma diffusing radially, as well as from the Z-line diffusing longitudinally. Without this diffusional restriction from the presence of SSM between the myofibril and sarcolemma, the Ca^2+^ level in the sub-sarcolemmal region increases compared with the interior region. The myofibrils next to the sarcolemma, but without SSM, develop approximately 15% greater force than other myofibrils. Inhomogeneous force development between sub-sarcolemmal and the interior region is not favorable for mechanical efficiency, and may even cause shearing within a cell. It is conceivable that SSM might serve to cancel the Ca^2+^ inflow through surface sarcolemma and average the intracellular [Ca^2+^] environment.

### Study limitations

McNary et al. (2011, 2012) demonstrated that stretching the myocyte reduces the total volume of the t-tubule system because the cell deformation shortens the t-tubules without changing their cross-sectional area. A volume decrease ejects extracellular fluid, encouraging changes in intra-t-tubular ion concentration due to excitation, thus affecting the electrical activity of the myocyte. In the present study, the t-tubule structure was modeled as a series of simplified triphasic truss elements, which permitted numerical solution within a practicable time frame. Modeling the t-tubule fluid with triphasic solid elements would have necessitated dividing them radially into at least five sections, which is not feasible for our computable mesh resolution. Although the current simplified t-tubule model can simulate the volume change caused by a pressure difference across the sarcolemma, resolving length changes is more difficult. In a future study, we plan to use a finer mesh to more realistically model the morphology of t-tubules (Hake et al., 2012; Das and Hoshijima, 2013) and account for their material properties. This will permit the evaluation of suction and ejection of t-tubule fluid on electrical activity.

In the present study, we have semi-quantitatively evaluated the intra-t-tubular diffusion coefficient and the relationship between t-tubular morphology, mitochondrial location, and the distribution of force development. Although the advancement of experimental technologies now enables us to visualize ions and molecules in living cells, its spatial resolution is still limited. Electron microscopy with proper molecular markers can provide high-resolution images, but they are snapshots of inactive cells. Detailed three-dimensional simulation enables studies beyond the limit of current experimental technologies.

### Conflict of interest statement

The authors declare that the research was conducted in the absence of any commercial or financial relationships that could be construed as a potential conflict of interest.

## Supplementary material

Supplemental Movie 1 **Temporal change in intracellular [Ca^2+^] with isosurfaces (4.0, 2.5, 1.0 mM)**. Time lapse images of the movie are shown in Figure 5A.

Supplemental Movie 2 **Temporal change in fluid velocity distribution in t-tubule under isometric contraction**. Time lapse image of the movie is shown in Figure 6A.
